# Presence of β-Turn Structure in Recombinant Spider Silk Dissolved in Formic Acid Revealed with NMR

**DOI:** 10.3390/molecules27020511

**Published:** 2022-01-14

**Authors:** Yu Suzuki, Takanori Higashi, Takahiro Yamamoto, Hideyasu Okamura, Takehiro K. Sato, Tetsuo Asakura

**Affiliations:** 1 Department of Applied Chemistry and Biotechnology, Graduate School of Engineering, University of Fukui, 3-9-1, Bunkyo, Fukui 910-8507, Japan; higasit@u-fukui.ac.jp (T.H.); bclbiospider0814@gmail.com (T.Y.); hokamura@u-fukui.ac.jp (H.O.); 2 Spiber Inc., 234-1 Mizukami, Kakuganji, Tsuruoka 998-0052, Japan; takehiro_sato@spiber.inc; 3 Department of Biotechnology, Tokyo University of Agriculture and Technology, 2-24-16, Nakacho, Koganei, Tokyo 184-8588, Japan

**Keywords:** spider silk, NMR, formic acid

## Abstract

Spider dragline silk is a biopolymer with excellent mechanical properties. The development of recombinant spider silk protein (RSP)-based materials with these properties is desirable. Formic acid (FA) is a spinning solvent for regenerated *Bombyx mori* silk fiber with excellent mechanical properties. To use FA as a spinning solvent for RSP with the sequence of major ampullate spider silk protein from *Araneus diadematus*, we determined the conformation of RSP in FA using solution NMR to determine the role of FA as a spinning solvent. We assigned ^1^H, ^13^C, and ^15^N chemical shifts to 32-residue repetitive sequences, including polyAla and Gly-rich regions of RSP. Chemical shift evaluation revealed that RSP is in mainly random coil conformation with partially type II β-turn structure in the Gly-Pro-Gly-X motifs of the Gly-rich region in FA, which was confirmed by the ^15^N NOE data. In addition, formylation at the Ser OH groups occurred in FA. Furthermore, we evaluated the conformation of the as-cast film of RSP dissolved in FA using solid-state NMR and found that β-sheet structure was predominantly formed.

## 1. Introduction

Silk protein is a promising resource in material science and medical applications because of its high biocompatibility, biodegradability, and excellent mechanical properties [1,2,3,4]. Silk proteins dissolved in suitable solvents can be processed to produce films, sponges, regenerated fiber, and non-woven mats. Among the solvents for typical *Bombyx mori* silk fibroin, commonly used organic solvents for processing [5] include hexafluoroisopropanol (HFIP) [6,7,8,9,10,11], hexafluoroacetone (HFA) [12], trifluoroacetic acid (TFA) [13], *N*-methyl morpholine *N*-oxide [14], and formic acid (FA) [13,15,16,17,18,19,20,21,22,23]. FA induces β-sheet formation of *B. mori* silk fibroin in the solid state; films prepared from the direct casting of the FA solution are insoluble in water without requiring further insolubilization treatments such as alcohol treatment and water annealing. The regenerated silk fibroin fiber prepared from FA or FA-CaCl_2_ used as spinning solvents has excellent mechanical properties.

Recently, spider dragline silk has attracted much attention as a resource for highly functional next-generation materials because of its remarkable mechanical properties, which are superior to most synthetic fibers [24,25,26,27,28,29,30,31,32,33,34,35,36,37,38,39,40,41]. In addition, upon exposure to water, the dragline silks contract up to 50% of their stretched length, a process known as supercontraction [42,43,44,45,46,47]. This process is accompanied by an increase in extensibility and a decrease in stiffness, resulting in rubber-like mechanical properties. The spider dragline silk consists of two proteins, major ampullated spidroin 1 (MaSp1) and spidroin 2 (MaSp2) [25,32,33,48,49], which differ in the amino acid sequence. The most apparent difference is that Pro residues exist exclusively in MaSp2 as Gly-Pro-Gly-X-X motif but are lacking in MaSp1. Since Pro acts to disrupt the secondary structure of protein, the mechanical and optical properties and supercontraction behavior are considerably different between the two proteins. Thus, the structure and dynamics of the GPGXX motif in MaSp2 will provide useful information regarding the extensibility and supercontraction mechanism of spider silk protein.

In our previous study [47], recombinant spider silk protein (RSP) with the sequences from ADF-3 silk protein (corresponding to MaSp2 protein) from the European garden spider *Araneus diadematus* was produced, and the structure and dynamics were studied using solid-state NMR in both dry and hydrated states. RSPs have been produced using a genetic engineering technique and could provide novel functional materials with the addition of functional groups for various applications [50,51]. Thus, RSP fiber could be produced with excellent mechanical properties from the FA solution used as a spinning solvent.

In this study, we analyzed the conformation of the RSP dissolved in FA using solution NMR and evaluated the conformation of a film prepared from RSP dissolved in FA using solid-state NMR. FA induces chemical modifications, particularly formylation of proteins [52,53,54,55]. Previous reports have shown the esterification of Ser and Tyr residues for peptides incubated in FA. Thus, the formylation of these residues in RSP was studied. The sequential assignment of repetitive sequences containing polyAla and Gly-rich regions with 32-residue length was performed using various multidimensional NMR measurements, to obtain ^1^H, ^13^C, and ^15^N chemical shifts for each residue. When comparing the chemical shift of each amino acid in the 32-residue sequence with the chemical shifts of typical secondary structures of proteins, no α-helix or β-sheet structures were observed in both the polyAla and Gly-rich regions. The dihedral angles were determined from the chemical shift using the program TALOS-N [56,57]. Some of the Gly-Pro-Gly-X motifs in the Gly-rich region had the dihedral angle of the type II β-turn structure [57]. This result was also supported by the cross-peak between ProHβ and GlyHN in the NOESY spectrum. In addition, ^15^N-{^1^H} steady-state NOE measurements indicated that the Gly-rich region was less flexible than the polyAla region. This result was in good agreement with the existence of some restricted structures of the Gly-Pro-Gly sequences in the Gly-rich region. Furthermore, we evaluated the secondary structure of the RSP films prepared by dissolving in FA and dried using solid-state NMR and determined that the polyAla region was mainly in β-sheet structure yet contained a random coil at the end groups of the polyAla region as-cast film without any insolubilization treatments. These results suggested that the RSP dissolved in FA was present in mainly random coil structure, while the Gly-rich region partially containing restricted structure. Finally, formylation at the Ser OH groups occurred in FA. These findings demonstrated that RSP has a certain unique conformation and modification in FA, which may contribute to the increased mechanical property of the regenerated RSP fibers.

## 2. Results and Discussion

### 2.1. ^1^H, ^13^C, and ^15^N Assignments of the Repetitive Domain in RSP in Formic Acid

**Figure 1:**
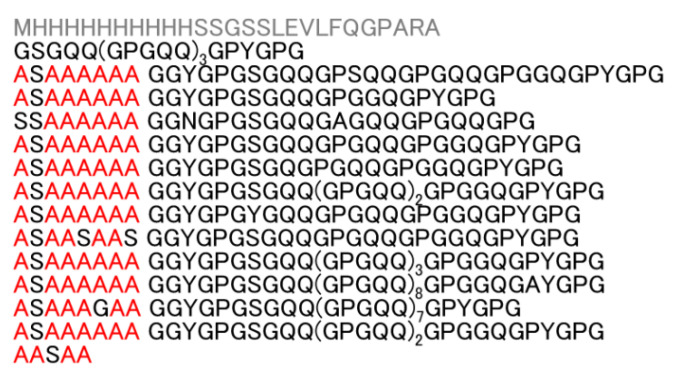
Primary structure of RSP based on the primary sequence of the MaSp2 silk component from the European garden spider, *Araneus diadematus*. Ala residues are in red; other amino acids, except the His-tag, are in black.

We analyzed RSP containing the amino acid sequence of the MaSp2 silk component from the European garden spider *A. diadematus* [47]. RSP is composed of 542 amino acid residues, with a molecular weight of 47,475 kDa. The amino acid composition is 37% Gly, 19% Gln, 15% Ala, 15% Pro, 6% Ser, and 4% Tyr. The primary structure of RSP (Figure 1) contains alternating polyAla and Gly-rich regions. The polyAla region mainly comprises seven Ala residues with one Ser residue inserted between the first and second Ala. The Gly-rich region consists of Gly, Gln, Pro, Ser, and Tyr. The structure and dynamics of RSP dissolved in FA were probed using solution NMR spectroscopy.

**Figure 2:**
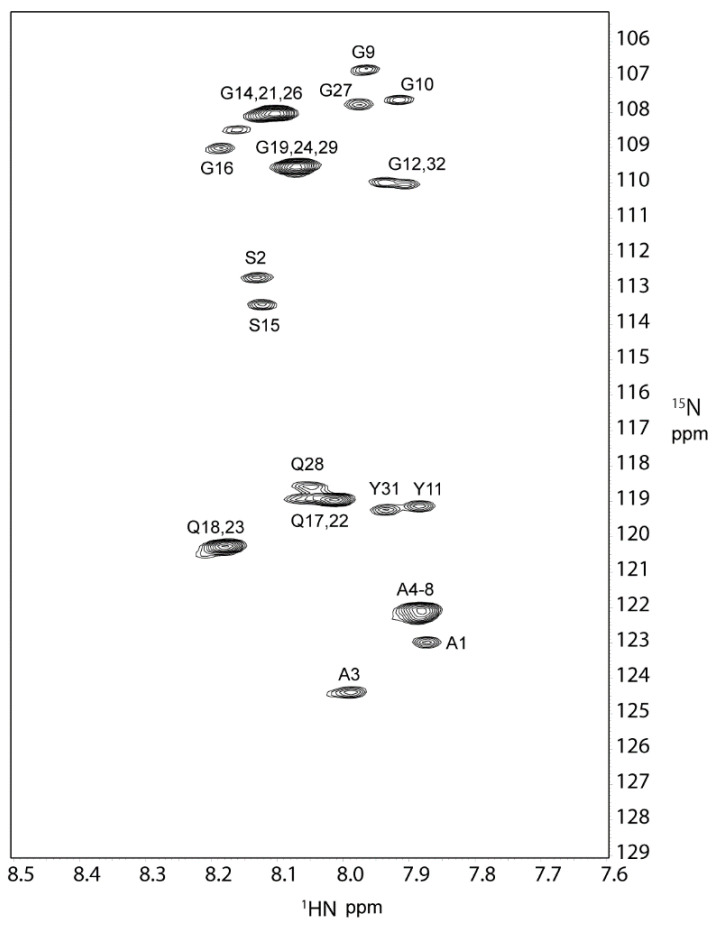
The 2D ^1^H-^15^N HSQC spectrum of U-[^13^C,^15^N] labeled RSP in FA at 298 K, 600 MHz. The 17 backbone cross-peaks are labeled with amino acid and residue number and listed in Table 1.

A 2D ^1^H-^15^N HSQC spectrum of RSP is shown in Figure 2. The spectrum exhibits 17 definite cross-peaks derived from backbone resonances. Five major residues, namely, Gly, Gln, Ala, Ser, and Tyr, are observed for RSP in FA. Each amino acid has multiple chemical environments, including seven Gly, three Gln, three Ala, two Ser, and two Tyr resonances. Cross-peaks derived from Pro constituting 15% of RSP were not observed in the ^1^H-^15^N HSQC spectrum since Pro is an imino acid and has no amide group in the polypeptide chain.

**Figure 3:**
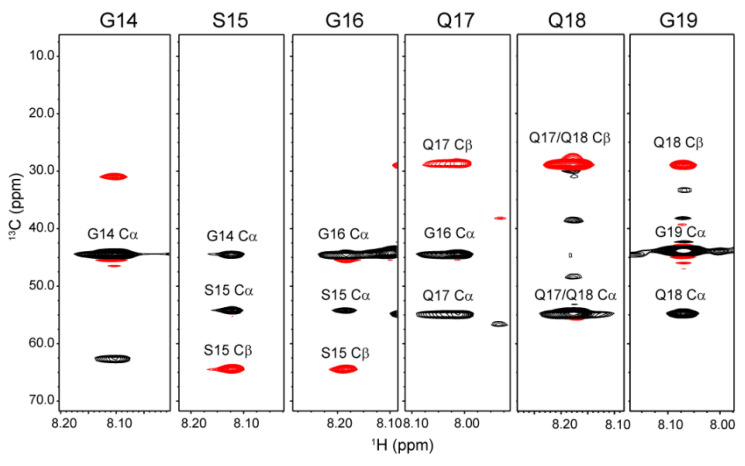
The strip plots of the 3D triple resonance HNCACB spectrum of U-[^13^C,^15^N] labeled RSP in FA collected at 298 K, 600 MHz. Selected ^15^N planes with ^1^H-^13^C correlations are displayed with antiphased ^13^Cα (black) and ^13^Cβ (red) cross-peaks from the corresponding amino acids and previous amino acids.

Spectral assignment was obtained using a combination of 2D and 3D data sets acquired at 298 K in FA, that is, ^1^H-^13^C HSQC, HNCO, HN(CA)CO, HN(CO)CA, HNCACB, CBCA(CO)NH, H(CCO)NH, and CC(CO)NH. Figure 3 shows a part of (Gly^14^-Gly^19^) HNCACB strip spectra used for making sequential assignments of backbone resonances. Sequential assignment was accomplished for the 32-residue repetitive sequence (ASAAAAAAGG^10^YGPGSGQQGP^20^GQQGPGGQGP^30^YG), which is the most abundant repetitive sequence in the RSP primary structure. The ^1^H, ^13^C, and ^15^N chemical shifts of each residue were determined and are shown in Table 1. Four Ala residues, from Ala^4^ to Ala^7^, in the polyAla region were assigned to the same chemical shift. The intensity of the peak assigned to Ala^4^-Ala^7^ was much higher than that of Ala^3^ and Ala^8^, indicating that peak assignment of Ala residues is highly probable. The sequence GQQGP can be seen twice in the sequence, and the three central residues, namely, Gln^17^-Gln^18^-Gly^19^ and Gln^22^-Gln^23^-Gly^24^, are assigned to the same chemical shifts.

### 2.2. Secondary Structure of the Repetitive Sequence in RSP in Formic Acid

The chemical shifts of the obtained repetitive sequences consisting of 32 residues were used to study the secondary structure. First, we used the chemical shift of each amino acid in the protein that formed a typical secondary structure (α-helix and β-sheet) as reported by Wishart et al. [58] We compared these reported chemical shifts with those of the assigned 32 amino acid residues. Since the reported chemical shifts are based on the data of the protein dissolved in water, the chemical shifts of the RSP dissolved in FA are likely to be affected by the solvent effect. Therefore, we used the ^13^C and ^15^N chemical shifts, which are less affected by solvent effects compared to ^1^H chemical shifts, which are more sensitive to solvent interactions. As a result, no residues with typical secondary structure formation tendency were found for the RSP repetitive sequence (data not shown). This was an expected result since the previous solution structures of native spider silk proteins dissolved in water before fiber formation and RSP in aqueous solution did not show the formation of typical α-helix or β-sheet structures. [36,59,60].

**Figure 4:**
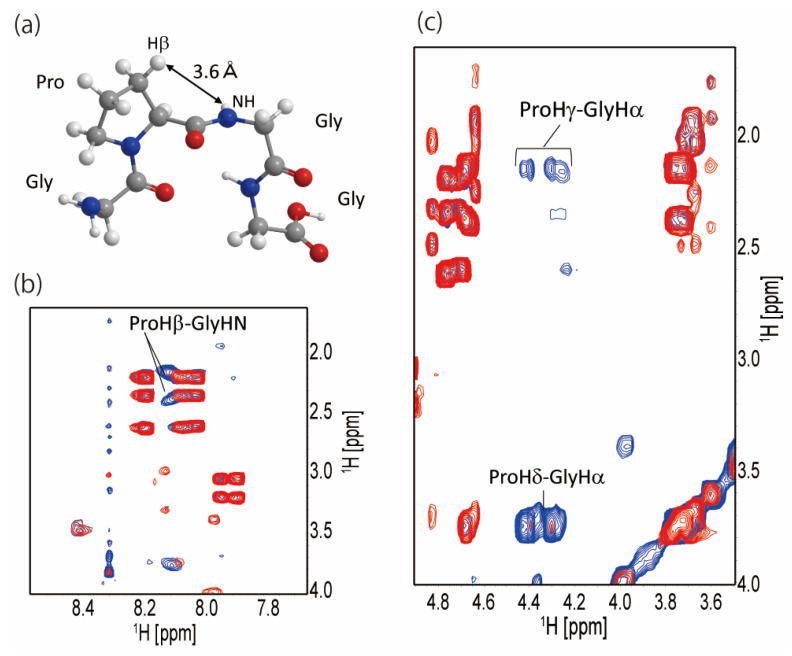
(**a**) Structural model of Gly-Pro-Gly-Gly motif formed typical type II β-turn structure; (**b**) superimposed spectra of ^1^H-^1^H NOESY (blue) and ^1^H-^1^H TOCSY (red) for RSP dissolved in FA. The NOESY cross-peaks between ProHβ and GlyHN whose distance is 3.6 Å illustrated in (**a**) are denoted. (**c**) Superimposed spectra of ^1^H-^1^H NOESY (blue) and ^1^H-^1^H TOCSY (red) for RSP dissolved in FA. The NOESY cross-peaks between GlyHα-ProHδ and GlyHα-ProHγ are denoted.

Next, we applied the program TALOS-N [56] to predict the dihedral angle from the chemical shifts. As a result, two of the four Pro-X in the 32 residues, Pro^20^-Gly^21^ and Pro^25^-Gly^26^, were found to be close to the typical dihedral angle of residues (i + 1) and (i + 2) of type II β-turn (Table 2). Then, we examined the cross-peaks observed in the NOESY spectra between the neighboring ^1^H nuclei in type II β-turn structure. Figure 4a shows the type II β-turn model structure of Gly-Pro-Gly-Gly, where the distance between Pro Hβ and Gly HN is 3.6 Å in type II β-turn. Figure 4b shows a part of the superimposed TOCSY and NOESY spectra; there is no TOCSY cross-peak between Pro Hβ and Gly HN, only NOESY cross-peaks are observed. It also indicated that the Pro-Gly sequence in the Gly-rich region partially forms type II β-turn. NOESY cross-peaks of ProHγ-GlyHα and ProHδ-GlyHα were also observed as shown in Figure 4c. These peaks indicate that the Gly-Pro-Gly sequence in RSP has some restricted conformation in FA.

Jenkins et al. reported 2D homo- and heteronuclear MAS solid-state NMR studies of the Gly-Pro-Gly-X-X motif in ^13^C/^15^N-Pro labeled *A. aurantia* dragline silk [61]. The data showed a secondary structure for the Pro residue in the motif similar to that of native elastin. Thus, they tentatively concluded that the Gly-Pro-Gly-X-X motif took a type II β-turn structure. The Gly-Pro-Gly-X sequence in the Gly-rich region of the RSP partially forms a type II β-turn in FA, which forms a structure similar to that of the spider silk protein MaSp2 in the silk fiber. Several studies have reported the structure of the repetitive sequences of spider silk proteins before fiber formation. The conformation of native spider silk proteins within the major ampullate (MA) gland was studied using HR-MAS NMR spectroscopy [59]. The conformation-dependent ^1^H and ^13^C chemical shifts showed that MaSp1 and MaSp2 of *Nephila clavipes* and *Araneus aurantia* were random coil in the MA gland. Moreover, solution NMR spectroscopy was used to characterize the backbone structure and dynamics of *Latrodectus hesperus* spider silk proteins in an intact MA gland [60]. The backbone dynamics of the spider silk proteins were obtained from ^15^N NMR relaxation parameters and ^15^N-{^1^H} steady-state NOE. These measurements revealed that the repetitive sequences of the spider silk proteins were highly flexible and unfolded. The native spider silk protein in the MA gland of *N. clavipes* was analyzed using solution NMR [15]. The ^13^C chemical shift showed that the polyAla region was neither α-helix nor β-sheet on the NMR time scale. Moreover, the Ala chemical shift of native spider silk protein dissolved in FA was consistent with that of native spider silk protein in the MA gland, indicating that the structure of spider silk protein in FA is similar to that in the MA gland.

### 2.3. Dynamics of the Repetitive Sequence in RSP in Formic Acid by ^15^N-{^1^H} Steady-State NOE Measurement

**Figure 5:**
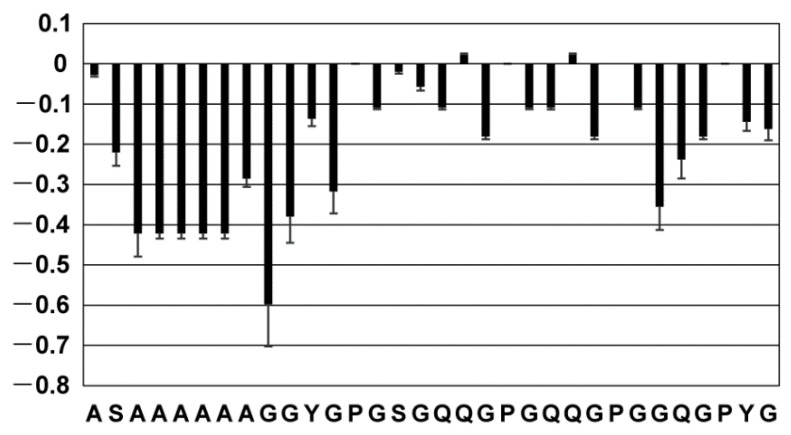
^15^N-{^1^H} steady-state NOE for each residue in the 32-residue repetitive sequence of RSP.

We measured ^15^N-{^1^H} steady-state NOE for RSP dissolved in FA. ^15^N NOE measurements provide information regarding the dynamics of backbone amide protons in proteins. The ^15^N NOE plot of the 32-residue repetitive sequence is shown in Figure 5. For residues in the SAAAAAAGG sequence, including the polyAla region, ^15^N NOE values were between −0.6 and −0.2. By contrast, for other residues, especially the GXGQQ (X = S, P) sequence, ^15^N NOE values ranged from −0.2 to 0, which were larger than those of the polyAla region. Basically, a higher value of ^15^N NOE suggests relatively lower flexibility, while a lower value suggests relatively higher flexibility. Therefore, a large ^15^N NOE value is obtained for folded polypeptides, and a small one is obtained for unfolded ones. Thus, the value of the ^15^N NOE rate of RSP indicated that the polyAla region is almost unfolded, and the GXGQQ in the Gly-rich region has limited flexibility compared to that of polyAla and its neighboring regions. The dihedral angles obtained from the chemical shift indicated that the Gly-Pro-Gly-X motif in the Gly-rich region partially forms a type II β-turn structure (Figure 4). This indicated that the Gly-rich region is not a completely random coil state but has a restricted steric structure, and the flexibility of the molecular chain is reduced compared to the random coil state. This result was in agreement with that of ^15^N NOE, which showed that the flexibility of the Gly-rich region is lower than that of the polyAla region, and that there is a β-turn structure in the Gly-rich region.

### 2.4. Solvent Effect of Formic Acid on RSP Structure

The results of secondary structure distribution obtained through ^1^H, ^13^C, and ^15^N chemical shifts and ^15^N NOE measurements revealed that RSP is mainly random coil conformation throughout the sequence and has a partial type II β-turn structure in Gly-Pro-Gly-X motifs in the Gly-rich region in FA. These results indicated that polyAla and Gly-rich regions are in different environments in FA. The hydrodynamic radii of silk fibroin were reported to be 139 and 19 nm in water and FA, respectively, which suggested that silk fibroin forms a more compact state in FA than in water [17]. Because FA is a carboxylic acid and readily interacts with polar groups, it interacts with amino acid side chains of polar groups such as CO, OH, COO^−^, and NH_3_^+^. Thus, Gly-rich regions that contain polar side chains, such as the amide group of Gln and hydroxyl groups of Ser and Tyr, are expected to interact with FA, whereas polyAla regions, which mostly comprise non-polar side chains, approach each other and form a hydrophobic core in the molecule. From this structural model of RSP in FA, we can explain why FA forms a stable solution with silk protein. The Gly-rich region with many polar groups contacts solvent molecules, and the polyAla region with a series of hydrophobic residues forms a hydrophobic core. Thus, inter-molecular associations and the subsequent aggregation caused by hydrophobic interactions are prevented. Even if some RSP molecules form a prefibrillar structure, the solvent molecules surrounding the RSP molecules suppress the aggregation-causing interactions between RSP molecules. Therefore, the solution of silk protein dissolved in FA is very stable.

Aluigi et al. reported the stability of keratin aged in FA [62]. They found that the fresh keratin solution dissolved in FA was not degraded at all, while the molecular weight of keratin dissolved in FA for two weeks was decreased partially. This result indicated that the fresh silk solution dissolved in FA is not degraded, although the silk that had been dissolved in FA for more than two weeks may decrease the molecular weight.

### 2.5. Formylation of RSP Occurred in Formic Acid

**Figure 6:**
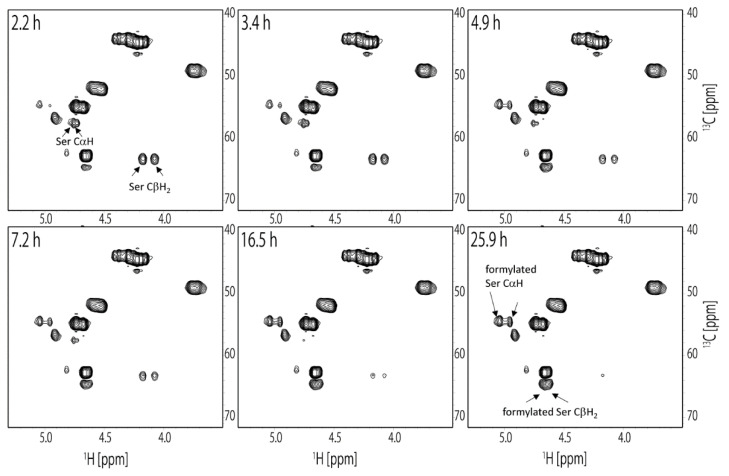
The selected spectra of the time course ^13^C HSQC experiments with RSP to observe the formylation in real time after the dissolution of RSP in FA. The elapsed time from start of the measurements is denoted at top left of each box. Peaks derived from unformylated and formylated Ser residues are denoted in the spectra.

Since FA is a known formylating agent, it is possible that the side chains of Ser and Tyr in RSP are formylated. In the previous report, the hydroxyl groups of the Ser residues in the β-amyloid peptide were formylated in FA [52,53,54,55]. Thus, we evaluated the formylation of RSP dissolved in FA by solution NMR and confirmed the formylation of the Ser side chain. The ^13^C HSQC spectra of RSP were measured several times continuously to observe the formylation in real time after the dissolution of RSP in FA. As a result, the Ser side chain was formylated, while the Tyr side chain was not. The chemical shifts of CH in the Ser side chain changed after the dissolution of RSP in FA, although the chemical shifts of hydrocarbons in the Tyr side chain did not change, even 36 h after dissolution. As shown in Figure 6, two sets of unformylated Ser CαH and CβH_2_ peaks corresponding to Ser^2^ and Ser^15^ residues in the repetitive sequence were observed in the first ^13^C HSQC spectrum. The ^1^H chemical shifts were (4.73 ppm, 57.5 ppm) and (4.76 ppm, 57.5 ppm) for CαH and (4.08 ppm, 63.3 ppm) and (4.17 ppm, 63.3 ppm) for CβH_2_, respectively.

**Figure 7:**
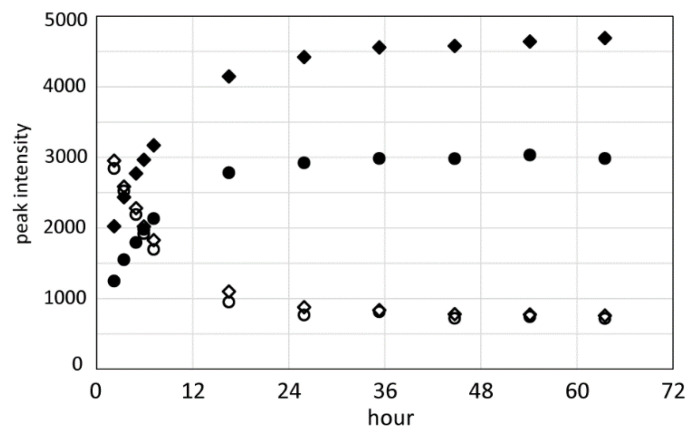
The plot of peak intensity for the CαH and CβH_2_ protons of unformylated and formylated Ser residues over time after the dissolution of RSP in FA. (● formylated Hα, ◆ formylated Hβ, ○ unformylated Hα, ◇ unformylated Hβ).

Intensity of these peaks gradually decreased with time. In the ^13^C HSQC spectrum measured 26 h after dissolution, new peaks corresponding to formylated Ser appeared at (4.95 ppm, 54.6 ppm) and (5.04 ppm, 54.5 ppm) for CαH and (4.62 ppm, 64.6 ppm) and (4.66 ppm, 64.6 ppm) for CβH_2_, respectively, as shown in Figure 6. Intensity of these peaks increased with time. The time-dependent changes of the peak intensities for the CαH and CβH_2_ of unformylated and formylated Ser residues are plotted in Figure 7. These measurements showed that most of the Ser residues in RSP were formylated within 36 h after dissolution in FA. The chemical shift of the Ser Cα peak shifted 2.9 and 3.0 ppm to a higher field, Ser Cβ peak shifted 1.3 ppm to a lower field, Ser αH peak shifted 2.2 and 2.8 ppm to a lower field, and Ser βH peak shifted 5.4 and 4.9 ppm to a lower field by formylation. Both Ser αH and βH protons shifted to a lower field by formylation. In the previous study, the formylation of the Ser side chain of β-amyloid peptide by FA treatment was evaluated by solution NMR. The peptides were dissolved in 88% FA and incubated overnight and dissolved in DMSO-*d*_6_ for NMR measurements.

The formylation resulted in a lower field shift of αH proton from 4.40 to 4.70 ppm and βH protons from 3.65 to 4.32 ppm. A lower field shifts of Ser αH and βH by formylation in our study were in good agreement with the results of the previous studies. These results indicated that the Ser side chain of RSP was almost formylated within 24 h in FA. Then, multidimensional solution NMR measurements for the evaluation of conformation and dynamics of RSP were conducted more than 24 h after dissolving RSP in FA.

### 2.6. Secondary Structure of the RSP Film in the Solid State Prepared from Formic Acid

**Figure 8:**
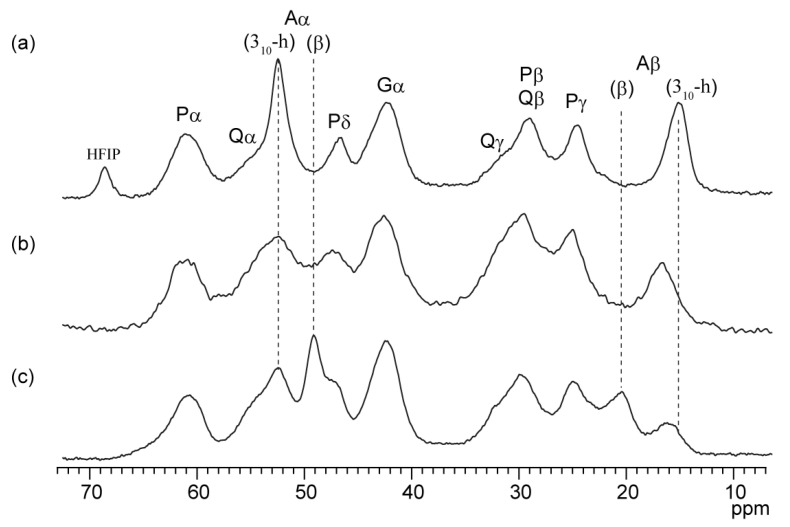
^13^C CPMAS NMR spectra of RSP films prepared using different solvents: (**a**) HFIP, (**b**) DMSO, and (**c**) FA with peak assignments. Ala Cα and Cβ are assigned secondary structures of a 3_10_-helix (3_10_-h) and a β-sheet (β), respectively.

The previous study showed that *B. mori* silk fibroin film prepared from FA solution is insoluble in water without further insolubilization treatments. FA induces silk fibroin to form a β-sheet structure in solid state. The as-cast film prepared from silk fibroin dissolved in FA is 38.9% crystalline, whereas the as-cast film prepared from an aqueous solution of silk fibroin is amorphous [16]. To clarify whether the film prepared by dissolving RSP in FA and drying forms a β-sheet structure, we evaluated the secondary structure of the film prepared from FA solution (FA-RSP) using solid-state NMR. The film prepared by dissolving RSP in HFIP (HFIP-RSP) and sponge prepared by dissolving RSP in DMSO (DMSO-RSP) were also evaluated to compare with FA-RSP. The ^13^C CPMAS NMR spectra of FA-RSP, HFIP-RSP, and DMSO-RSP are shown in Figure 8. Peaks were assigned based on a previous study on ^13^C CPMAS NMR of the film prepared from RSP dissolved in HFIP [47] and the reference of secondary structure dependence of the chemical shifts [56]. The secondary structure tendency of Ala residues in the polyAla region was evaluated using the Ala Cβ and Cα chemical shifts. The Ala Cβ peaks were observed at 15.0 ppm for HFIP-RSP, 16.5 ppm for DMSO-RSP, and 16.0 and 20.3 ppm for FA-RSP. HFIP promotes helix formation; the chemical shifts of the Ala Cβ peak in HFIP-RSP revealed that it forms mostly 3_10_-helical structures [9,63]. DMSO treatment tends to turn fibroin into the random coil structure; the Ala Cβ chemical shift of DMSO-RSP revealed that in RSP, Ala residues mainly formed random coil structures. The Ala Cβ peak chemical shift of FA-RSP indicated that FA-RSP mainly forms β-sheet structures. This was consistent with the results that insoluble films are obtained when silk fibroin is dissolved in FA. The chemical shift of the Ala Cα peak also reflected the secondary structure as well as the chemical shift of Ala Cβ. In the case of Ala Cα, contrary to Ala Cβ, the structure is 3_10_-helix, random coil, and β-sheet from a lower field to a higher field. HFIP-RSP gives a sharp peak at 52.3 ppm, which indicated that it is mainly a 3_10_-helix structure. FA-RSP also gives a sharp peak at 49.0 ppm, which indicated that it is mainly a β-sheet structure. The DMSO-RSP has a peak top at the same chemical shift as the HFIP-RSP, but the peak is broader, and the Gln Cα peak overlaps the lower field side. Therefore, the actual chemical shift of the Ala Cα peak for DMSO-RSP is expected to be slightly smaller than 52.3 ppm, indicating that the polyAla region of DMSO-RSP is mainly random coil structure.

## 3. Materials and Methods

### 3.1. Preparation of Recombinant Spider Silk Protein, RSP

RSP, with the amino acid sequence encoded by the ADF-3 fibroin gene of *A. diadematus*, was produced using *Escherichia coli* and purified using a Ni column [47]. A His-tag was attached to the *N*-terminus of the amino acid sequence for sample purification. Uniformly labeled (^13^C, ^15^N) RSP was also produced by using M9 minimal medium containing (2 g/L) ^13^C-glucose and (1 g/L) ^15^N-ammonium. Figure 1 shows the amino acid sequence of RSP.

### 3.2. Solution NMR Measurements

The RSP powder was dissolved in formic acid-d_1_ (Cambridge Isotope Laboratories, Inc., Tewksbury, MA, USA) to a concentration of 0.5 mM and stored in a 5 mm Shigemi microtube. NMR experiments were performed on a Bruker (Billerica, MA, USA) AVANCE III HD (600 MHz) equipped with a QCI cryogenic probe and JEOL (Tokyo, Japan) Resonance ECZ500 spectrometer at 298 K. The assignments of the ^1^H, ^13^C, and ^15^N peaks to the residues were accomplished using ^1^H-^15^N HSQC, ^1^H-^13^C HSQC, HNCO, HN(CACO), HN(CO)CA, HNCACB, CBCA(CO)NH, HCCONH, and CCCONH. ^1^H-^1^H NOESY and ^1^H-^1^H TOCSY spectra were also recorded. All spectra were processed using NMRPipe [64] and analyzed using MagRO-NMRView [65]. TMS proton signal at 0 ppm was used as a chemical shift reference for ^1^H signals. ^13^C and ^15^N chemical shifts were indirectly referenced by using ^1^H chemical shift. Furthermore, ^15^N-{^1^H} steady-state NOE values were measured with a proton saturation of 3 s within a relaxation delay of 4 s for analyzing backbone dynamics. Dihedral angle constraints for the main chain were derived from database analysis of the chemical shifts of the backbone atoms using the protein backbone dihedral angle prediction program named TALOS-N [56]. Non-labeled RSP was dissolved in formic acid-d_1_ and the ^1^H-^13^C HSQC spectrum was observed using a JEOL (Tokyo, Japan) ECZ500 NMR spectrometer to examine the formylation at the Ser OH group.

### 3.3. Solid-State NMR Measurements

RSP powder was dissolved in FA, and the solution was dried for 5 d at 25 °C to prepare as-cast films. RSP powder was dissolved in 2 M LiCl-DMSO at 60 °C, and the solution was diluted twice with 7 M urea. Then, it was dialyzed with distilled water for 3 d and lyophilized. ^13^C CPMAS NMR spectra of RSP prepared using FA were recorded using the JEOL (Tokyo, Japan) ECA600 II NMR spectrometer, with a 3.2-mm MAS probe and an MAS frequency of 10 kHz. The sample was inserted into a zirconia rotor. Experimental parameters for the ^13^C CPMAS NMR experiments were 2.3 μs ^1^H 90° pulse, 3 ms ramped CP pulse with 108 kHz rf field strength, TPPM ^1^H decoupling during acquisition, 3 s recycle delays, 1024 data points, and 15 k scans. The ^13^C chemical shifts were calibrated externally through the methylene peak of adamantane observed at 28.8 ppm with respect to TMS at 0 ppm.

## 4. Conclusions

This study reports the conformation and dynamics of RSP dissolved in formic acid using solution NMR. ^1^H, ^13^C, and ^15^N chemical shifts of the 32-residue repetitive sequence were determined using a combination of multidimensional NMR measurements. Chemical shift evaluation revealed that RSP is mainly random coil conformation with partially type II β-turn structure in the Gly-Pro-Gly-X motifs of the Gly-rich region in FA. In addition, the formylation at the Ser OH groups occurred in FA. Furthermore, solid-state NMR measurements of FA-RSP revealed that RSP in the film made by dissolving in FA forms β-sheet structure without any insolubilization treatment. This suggests that in FA, unlike other organic solvents, silk forms a soluble prefibrillar structure in solution and retains a structure that facilitates the formation of β-sheet crystalline domains.

## Figures and Tables

**Table 1:** ^1^H, ^13^C, and ^15^N chemical shifts (in ppm) of amino acid residues in the 32-residue repetitive sequence of RSP in FA. Amino acids are described with three letter codes.

Residue Number	Amino Acid	N	NH	HA	CA	CO	CB
1	Ala	123.0	7.87	4.61	51.9	176.9	18.2
2	Ser	112.6	8.13	4.91	54.5	172.2	64.1
3	Ala	124.4	7.99	4.51	52.0	176.6	17.9
4~7	Ala	122.0	7.88	4.51	51.8	177.1	18.2
8	Ala	122.2	7.89	4.61	51.8	177.1	18.2
9	Gly	106.9	7.97	4.18	44.5	173.9	-
10	Gly	107.6	7.91	4.18	44.4	173.0	-
11	Tyr	119.1	8.01	4.87	56.9	175.1	38.3
12	Gly	110.0	7.93	-	43.8	171.2	-
13	Pro	-	-	4.73	62.8	176.7	31.0
14	Gly	108.0	8.10	4.28	44.5	173.4	-
15	Ser	113.4	8.12	5.04	54.3	172.8	64.5
16	Gly	109.0	8.18	4.18	44.7	173.3	-
17	Gln	118.9	8.01	4.69	54.9	174.7	28.7
18	Gln	120.3	8.18	4.74	54.8	174.8	28.9
19	Gly	109.6	8.07	-	43.9	171.2	-
20	Pro	-	-	4.69	62.8	176.7	31.0
21	Gly	108.0	8.10	4.28	44.5	173.4	-
22	Gln	118.9	8.01	4.69	54.9	174.7	28.7
23	Gln	120.3	8.18	4.74	54.8	174.8	28.9
24	Gly	109.6	8.07	-	43.9	171.2	-
25	Pro	-	-	4.69	62.8	176.7	31.0
26	Gly	108.0	8.10	4.28	44.5	173.4	-
27	Gly	107.7	7.98	4.25	44.5	173.4	-
28	Gln	118.6	8.04	4.74	54.8	173.4	28.6
29	Gly	109.6	8.07	-	43.9	171.2	-
30	Pro	-	-	4.63	62.6	175.6	30.9
31	Tyr	119.2	7.93	4.92	56.6	175.0	38.1
32	Gly	110.0	7.91	-	43.8	171.2	-

**Table 2:** Dihedral angle of the (i + 1) and (i + 2) residues of the four Pro-Gly motifs in the repetitive 32-residue sequence calculated by TALOS-N together with typical dihedral angle of type I β-turn and type II β-turn.

	Φ_i_	Ψ_i_	Φ_i + 1_	Ψ_i + 1_	Φ_i + 2_
Type I β-turn			−65 ± 12	−24 ± 13	−93 ± 16
Type II β-turn			−61 ± 13	136 ± 11	80 ± 16
G^12^P^13^G^14^	105	−177	−65	148	−124
G^19^P^20^G^21^	−88	−180	−63	146	73
G^24^P^25^G^26^	−87	−178	−66	148	111
G^29^P^30^Y^31^	132	−175	−70	151	−85

## Data Availability

The data presented in this study are available on request from the corresponding author.

## Sample Availability

Samples are not available from the authors.
